# Meeting report: “Depression and Anxiety Spectrum disorders: from basic science to the clinic and back”

**DOI:** 10.1186/2045-5380-3-6

**Published:** 2013-03-05

**Authors:** Suzanne N Haber, Ziad Safadi, Mohammad R Milad

**Affiliations:** 1 Department of Pharmacology and Physiology, University of Rochester School of Medicine and Dentistry, Rochester, NY 14642, USA

## Abstract

In March, 2012 we held the first Mideast conference on “Depression and Anxiety Spectrum disorders: from basic science to the clinic and back”, at the University of Amman, Jordan. This event brought together both clinical and basic scientists with expertise in depression and anxiety spectrum disorders. The meeting took place in a large lecture hall at the University of Jordan Medical School. The audience included faculty, residents, and students. The Dean of the Medical School opened the meeting, welcoming the guest speakers and participants.

## 

Anxiety disorders are generally characterized by irrational and excessive fear along with chronically elevated levels of anxiety. The trigger for the inappropriate fear and anxiety may be clear and well-defined in some of the disorders, such as specific phobias, posttraumatic stress disorder, or social anxiety disorder, or may be unspecific or not defined in other disorders such as panic disorder and generalized anxiety disorder. Depression is commonly characterized by lost of interest in engaging in daily activities, feelings of hopelessness, lack of energy and concentration, and feelings of incompetence. Depression and anxiety share common clinical features. Between 1/3 and 2/3 of patients who suffer from major depression experience some form of anxiety as part of their illness [1]. Moreover, the prevalence of major depression in patients with anxiety disorders is significantly higher than in the general population. Co-morbidity between anxiety and depression is common [2,3]. This relationship extends to family studies. Twin studies suggest shared genetic vulnerability to depression and anxiety. Effective treatments for depression and anxiety also notably overlap: medications acting on monoaminergic systems, and psychotherapies based on fear extinction and other methods geared toward reducing avoidance and increasing goal-directed, rewarding activities. Importantly, there is also a common neural network associated with these diseases. This network is central to the pharmacological and behavioral approaches for treatment and the hypotheses that drive the research of the underlying pathophysiology. The network includes the prefrontal cortex, specifically the orbital and anterior cingulate cortices, the ventral striatum, amygdala, and the brainstem catacholaminergic systems, particularly dopamine and serotonin [4].

Although there are no ‘hard’ data available for most Middle East countries [5], the population afflicted with these diseases is growing according to our colleagues practicing in there. Moreover, these diseases are massively under diagnosed and under treated. This meeting provided a unique opportunity to discuss the basic and clinical science that focus on the biological underpinnings of these mental health disorders.

The goal of the meeting was three-fold: first, to bring together experts on the pathophysiology and treatment of depression and anxiety spectrum disorders in a culturally diverse environment, where such disorders are prevalent, but exposure to cutting-edge therapies and research is limited; second, to explore the directions in which science is driving this field by presenting data on experimental and therapeutic approaches to understanding and treating these syndromes; and finally, to address the possibilities for future training and research in the region. The last goal was particularly important, as this meeting provided a forum to explore needs of the region and possible future collaborations.

There were two general topics, with sub-topics within each. The first general topic, “Biological basis of mood and anxiety disorders: A clinical and basic science overview”, focused on an overview of where the field is now, including a brief history. This session started with 2 general overviews, one on clinical aspects of mood and anxiety disorders (Dr. Greenberg) and a general discussion of the reward circuit that underlies these disorders (Dr. Haber). These general introductions were followed by talks focused specifically on state of the art clinical and basic science approaches. Dr. Eskandar gave an overview of neurosurgical and physiological studies in both animal and human studies; Dr. Akil gave an overview of the underlying biology associated with stress and depression; and Dr. Dougherty discussed using neuroimaging approaches to understanding these diseases. The second topic, ”Biological basis of mood and anxiety disorders: New advances”, focused on ongoing research. Dr. Moghaddam discussed reward and anxiety processing in adolescents, their vulnerability to disease, and goals for prevention; Dr. Aston-Jones talked about ongoing studies focused on the locus coeruleus; Dr. Greenberg discussed the clinical and basic science perspective of obsessive-compulsive disorder while Dr. Grace focused on animal models of the disease. Finally, the last afternoon of the meeting was dedicated to trauma and fear, with Dr. Pitman discussing stress, anxiety, and trauma, Dr. Milad focusing in fear and fear extinction, and Dr. Quirk discussing the neurobiology of fear learning. In addition there were some specialized talks, giving a perspective from the regions. These included a presentation from Dr. Jadiry on the prevalence of anxiety and mood disorders. Dr. Sarhan summarized cognitive behavioral therapy of Islamic religious OCD, and Dr. BaniMustafa on depression in primary care patients. We summarize the talks below.

The meeting was very well received and prompted numerous thoughtful questions and discussions. Students made comments such as “This meeting has completely changed my thinking and now I want to do research in neuroscience.” Faculty comments were equally positive; “The meeting was a success by all standards this is not personal view alone but the feedback I got from meeting people who attended.” Several also indicated how important it was to have this meeting. “The meeting about anxiety and depression was a great opportunity for clinicians and neuroscientists to meet to discuss the latest research in the field, which is not given the attention it deserves in undergraduate and postgraduate medical education taking in consideration that more than half of those who suffer from anxiety and depression go unrecognized and never receive treatment.” This impact on medical and graduate students is perhaps the most important legacy of the symposium.

Topic 1: Biological basis of mood and anxiety disorders: A clinical and basic science overview.

### 1A. Clinical overview and the reward circuit

Dr. Benjamin D. Greenberg from the Departments of Psychiatry and Human Behavior in the Alpert Medical School of Brown University gave the first talk, entitled “*Overview of mood & anxiety disorders”.* Dr. Greenberg emphasized several main points: that patients with the same categorical diagnosis as defined in the DSM system are in fact heterogeneous; that longitudinal stability of most diagnoses is poor; and that co-occurrence (“comorbidity”) of diagnoses tends to be the rule. For example, one- to two-thirds of individuals with an anxiety disorder will have a lifetime mood disorder; conversely, two-thirds of individuals who develop major depression or dysthymia will also have an anxiety disorder during their lifetimes.

In this context, he noted that the development of medication treatments in psychiatry was initially focused on how medications affected core clinical features of affected individuals as much as (or more than) than on treating illnesses as sepble entities. Such core features include an impaired ability to respond to reward after motivated behaviors, or positive emotional and social experiences (anhedonia). He proposed that an essential step towards making it possible for clinicians to target specific treatments to those individuals most likely to benefit from them, was to seek to understand behavioral and brain mechanisms underlying how effective behavioral and medication treatments may change such clinical features. The same approach should allow refinement of existing therapies, and development of new ones. Moreover, progress in this domain should enhance public understanding of psychiatric illnesses as disorders of the brain and behavior, which, in time, should reduce stigma and encourage treatment seeking, across cultures.

Dr. Suzanne Haber Professor at the Department of Pharmacology and physiology at the University of Rochester Medical School gave a presentation entitled, “*Understanding the Reward Circuit: Implications for Depression and Anxiety Spectrum Disorders*”. The presentation began with a brief overview of the background and the history of research of the reward system, followed by describing the reward network. She noted that while many brain regions respond to reward, central to the network are structures embedded within the cortico-basal ganglia system and include, the orbital, and anterior cingulate cortices, the ventral striatum, the ventral tegmental, midbrain dopamine neurons. In addition, other structures including the dorsal prefrontal cortex, amygdala, hippocampus, thalamus, lateral habenular n., and specific brainstem structures, such as the pedunculopontine n., and the raphe n., are key components in regulating the reward circuit. Connectivity between these areas forms a complex neural network. This network mediates different aspects of reward processing, including value, magnitude, and anticipation. Dr. Haber discussed how delineating these neurocircuits are key elements for understanding anatomical substrates of psychiatric diseases.

Classically, the cortico-basal ganglia system has been functionally divided into septe and segregated circuits. However, while the topographic organization of cortico-striatal projections are well documented, there is increasing evidence that there are also regions of interface between terminals from different cortical areas, suggesting functional integration to link motivation to behavioral output, critical for developing appropriate action plans. Dr. Haber demonstrated two ways in which cortico-basal ganglia circuits can integrate this information across functional circuits, through cortico-striatal projections, and through striatal-midbrain-striatal connections.

Projections from the cortical regions associated with motivation terminate in specific striatal regions, but also converge with terminals from associative areas. This complex interweaving and convergence provides an anatomical substrate for modulation between these motivation and cognition. This creates activation sites that may be particularly important for incentive-based learning. In addition, there is an interface between different functional striatal regions via the midbrain DA cells. Dr. Haber demonstrated how this interface is organized in an ascending spiral interconnecting different functional regions of the striatum. This anatomical arrangement creates a hierarchy of information flow, and provides an anatomical basis for the limbic/cognitive/motor interface via the midbrain dopamine neurons.

Dr. Haber ended her talk by describing translational studies, linking animal anatomical studies with neuroimaging. Diffusion tensor imaging (DTI) is increasingly used to develop models of prefrontal connections, to examine chances in white matter (WM) integrity associated with disease, and to target surgical interventions, including deep brain stimulation for psychiatric disease. Dr, Haber described how her lab uses nonhuman primate tracing studies coupled with 3-D reconstructions of WM bundles to delineate the rules frontal cortex axons follow to reach their targets. She then demonstrated that human prefrontal WM pathways follow these organizational rules. Taken together, these data provide the foundation for dividing white matter bundles into functional components and for predicting what is likely to be carried at different points through each bundle. Understanding this organization is key for determining specific connections that are likely to be captured at different neurosurgical targets.

### 1B. What we can learn from clinical and basic science

Dr. Emad Eskandar, MD, from the Department of Neurosurgery Massachusetts General Hospital presented two talks at the symposium. The first talk was entitled “Physiological Mechanisms of Basal Ganglia Disorders”. In this presentation, Dr. Eskandar presented data on the role of the basal ganglia and movement psychiatric disorders. First, Dr Eskandar gave a description of the relevant anatomy and physiology, followed by a reviewed surgical treatment options such as deep brain stimulation. This portion of talk discussed the rational for treatment and the key technical aspects of the surgery. Finally, Dr. Eskandar reviewed a number of prospective randomized clinical trials illustrating the benefits and limitations of the surgical treatments. Later in the symposium, Dr. Eskandar gave a talk entitled “Insights from Human and Primate Neurophysiology” which focused on the important role played by basic physiologic research in translating scientific findings into useful treatments for human disorders.

Dr. Huda Akil Professor of Neuroscience & Psychiatry and Director of the Molecular and Behavioral Neuroscience Institute, at the University of Michigan. She began her talk entitled “The Neurobiology of Stress and Depression: Animal Models and Human Studies” by noting that her late father, Dr. Fakher Akil (Akel) was one of the founders of the University of Jordan almost 50 years ago, and a member of the faculty of its Psychology Department when it was first established. She shared photographs of Dr. Akil with His Majesty the late King Hussein and other founding members at the dedication of the University of Jordan, and she noted that this gave this meeting a very special meaning for her.

Dr. Akil emphasized the burden of depression around the world, and the underlying complexity of the illness, as it has many genetic and environmental causes, converging to disturb the functioning of brain circuits that control our responses to stress and our short term and long-term moods. But currently available treatments for depression only work for a subset of patients, and at least a third of depressed individuals do not respond to any of the current treatments and become increasingly more depressed, some of them ending their lives tragically. So, how can science discover the biological causes of depression and identify new molecules that may become targets for new types of antidepressant drugs?

Dr. Akil described how her laboratory has created animal models of increased vulnerability to depression. One model uses a genetic approach in mice, and shows that increasing the activity of a gene that responds to stress only in the brain, and only during early development, is sufficient to produce an anxious and emotionally labile animal that serves as an excellent model for bipolar (manic-depressive) disorder. Thus, the stress the glucocorticoid receptor gene, GR, may be a good target for treatment in some patients.

Another approach was to create models of “temperament” by developing genetically bred lines of rats that are either prone to anxiety and depression-like behaviors or resistant to stress. These animals show characteristics of “internalizing disorders” versus “externalizing disorders”. Studies of developmental, molecular and neural bases of these differences have already yielded many surprising discoveries.

Dr. Akil then spoke about a complementary approach, where she and her collaborators begin their search in the brains of patients who had died when severely depressed compared to non-depressed controls. Using broad genetic and gene expression strategies, they identify molecules in specific brain regions that differentiate the severely depressed brains. Then, they return to the animal models to examine the function of these molecules. Using this strategy, they discovered that a family of growth factors, the FGF system, is severely disrupted in major depression. Studies in the animal models show that FGF2, which is low in depressed human brains, serves as a natural anti-depressant and anti-anxiety agent that is present in all brains, but differences in its activity can modify mood. Thus, animals that are prone to anxiety and depression, like depressed humans, have low levels of FGF2. Social stress also lowers the levels of FGF2. Importantly, replacing FGF2 in the brains of anxiety-prone animals reverses these tendencies, acts as an antidepressant and increases their activity and exploration. Thus FGF family has become a new target for studies of depression and as a potential target for developing new types of antidepressants.

In sum, combining basic science research and human clinical studies, and using genetic, molecular, anatomical and behavioral approaches are yielding exciting new insights into the causes of depression and hold promise for developing new treatment strategies for these stress-related brain disorders.

Dr. Darin D. Dougherty from the Department of Psychiatry at Massachusetts General Hospital and Harvard Medical School gave a presentation entitled, *“Neuroimaging, what we know and what we need to know”*. The presentation began with a brief overview of studies utilizing modern neuroimaging technology that have helped to elucidate regarding the pathophysiology of major depressive disorder (MDD) and obsessive-compulsive disorder (OCD). In brief, when compared to healthy volunteers, MDD appears to be associated with decreased resting activity in more dorsal neocortical brain regions associated with cognitive processes and increased resting activity in more ventral limbic and plimbic brain regions associated with the neurovegetative symptoms common to MDD. In addition, during neuroimaging studies that use a variety of activation pdigms in subjects with MDD and healthy volunteers, these dorsal regions are hyporeactive during cognitive tasks and these ventral regions are hyperreactive during affective tasks in subjects with MDD. Lastly, most of these abnormalities are attenuated following successful treatment (with either pharmacotherapy or psychotherapy) of the major depressive episode. In contrast, when compared to healthy volunteers, subjects with OCD exhibit greater resting activity in a specific cortico-thalamo-striato-cortical pathway that includes the orbitofrontal cortex (OFC), thalamus, and head of the caudate nucleus and these abnormalities can be amplified during pdigms that temporarily worsen OCD symptomatology. As is the case with MDD, these abnormalities are attenuated following successful treatment (with either pharmacotherapy or psychotherapy) of OCD.

After describing the rudimentary pathophysiology of both MDD and OCD, Dr. Dougherty described initial attempts to understand the effects of deep brain stimulation (DBS) at the ventral capsule/ventral striatum (VC/VS) target on these circuits using functional neuroimaging techniques. Because of the presence of metal in the DBS leads, most studies to date have used positron emission tomography (PET). The use of ^15^O isotopes (either in CO_2_ in room air or in water) allows for the study of acute changes in blood flow. Interestingly, studies using PET and ^15^O in subjects with VC/VS DBS for OCD have found acute blood flow changes in all nodes (OFC, thalamus, caudate) of the very circuit implicated in the pathophysiology of OCD. Initial data from studies using ^18^FDG and PET in subjects with VC/VS DBS for OCD to identify chronic (e.g., following 3-6 months of DBS) changes are suggesting that the same brain regions that exhibit these acute changes in blood flow continue to demonstrate changes from baseline following chronic treatment with VC/VS DBS. Compble studies in subjects receiving VC/VS DBS for MDD are ongoing. Dr. Dougherty concluded with a review of potential future directions, including using baseline neuroimaging data to identify individuals who are more likely to respond following DBS and to possibly tailor electrode targeting.

Topic 2: Biological basis of mood and anxiety disorders: New advances.

### 2A. Depression and Stress

Dr. Bita Moghaddam from the department of neuroscience at University of Pittsburgh gave a talk entitled “ Neuronal mechanisms for behavioral and psychiatric vulnerability in adolescents”. Dr. Moghaddam discussed how adolescents often respond differently than adults to the same salient motivating contexts, like peer interactions and stressors. Adolescence to early adulthood is also a period of psychiatric vulnerability and the typical time of symptom onset for several psychopathologies, including depression, addiction and schizophrenia. Thus, while the great majority of animal and human research related to these illnesses has focused on adults, research findings on the adolescent to early adulthood developmental age range may be more relevant to understanding the biological mechanisms that may contribute to the disease process and prevention. Adolescence encompasses puberty, a time of reproductive development, but also characterized by a myriad of non-reproductive socio-behavioral changes. Concomitant with these behavioral changes, adolescents undergo significant neurodevelopmental changes, including axonal growth, myelination and synaptic pruning, and changes in modulatory regulation of prefrontal cortex activity. Despite its importance, this stage of development remains understudied at mechanistic levels that are relevant to motivated behavior. This has been in part due to technical limitations associated with adolescent human and animal models. We believe that age-related changes in the ways salient stimuli are processed in key brain regions could underlie the unique predilections and vulnerabilities of adolescence. Because motivated behavior is the central issue, it is critical that age-related comparisons of brain activity be undertaken within motivational contexts. Dr. Moghaddam presented single neuron and large-scale electrophysiological data showing that the pattern of activation of cortico-striatal circuits, and the inhibitory-excitatory balance in these circuits, is different during motivated behavior in adolescent rats. These data indicate that there is reduced efficiency in the processing of cortical neural activity and related behaviors in adolescents. Furthermore, during this developmental period, regions critically involved in learning and habit formation are highly responsive to reward. This work highlights neuronal processing differences in adolescence that suggest possible mechanisms contributing to the vulnerability that this period presents for psychiatric illnesses.

Dr. Gary Aston-Jones, from the Medical University of South Carolina gave a talk entitled “The cortex in context: Locus coeruleus, optimal performance, and maximal utility” Optimal performance on a specific task requires selective attention to filter out extraneous stimuli and behaviors. It is also important, however, to disengage from the current task and find a new behavioral goal if motivation or the stimulus context changes. This tension between selective attention and the search for a new task in an altered context has been characterized as the exploitation-exploration tradeoff, and is critical for adaptive behavior. LC neurons exhibit two patterns or modes of activity in monkeys performing cognitive tasks: a *phasic mode* in which these cells are transiently activated selectively following decisions that were made, and a *tonic mode* when these cells have elevated tonic levels of discharge but no phasic responses. We hypothesize that the phasic mode of LC activity promotes selective behavioral responding to task-defined stimuli (selective attention), whereas the tonic mode produces a broader sampling of stimuli in the environment (search for new task, high behavioral flexibility). Both of these modes serve to increase the utility of behavior: The phasic mode increases utility on a short time-scale by facilitating selective responding in the task at hand, whereas the tonic mode increases long time-scale utility by promoting the identification of a new task that is adaptive in a changed context. In view of this functional framework, impairments of LC activity and ability to fluidly and adaptively change activity modes can produce deficits involved in several mental disorders, including attention deficit-hyperactivity disorder (persistent tonic mode), autism (persistent phasic mode), or sleep disorders and depression (persistently low activity).

### 2B. OCD and Anxiety

Dr. Benjamin D. Greenberg from the Departments of Psychiatry and Human Behavior in the Alpert Medical School of Brown University gave second talk entitled OCD*, clinical and basic science perspectives”.* He discussed one specific diagnosis, obsessive-compulsive disorder (OCD). He presented data on the population prevalence, course, clinical features (including subtypes based on differing symptom profiles) and the full range of current treatment approaches from behavior therapy to neurosurgery. The presentation of the range of clinical features was intended to give clinicians and others in the audience assistance in diagnosing OCD, the prevalence of which has historically been under-appreciated throughout the world. While current epidemiological information suggests that OCD, which typically has its onset in childhood or adolescence, affects about one percent of adults in any given year, many affected individuals fail to seek treatment, or may do so only after a long delay. As for other psychiatric illnesses, accurate diagnosis is often best accomplished via obtaining information from multiple sources, including potentially family members.

Then, after describing conventional behavioral and medication treatments, Dr. Greenberg turned to the subset of OCD sufferers with disabling, “intractable” illness, in whom surgical approaches might be considered. Determining if the strict selection criteria for, e.g., deep brain stimulation (DBS) are met requires exhaustive presurgical evaluations in multiple domains of symptoms, comorbidities, cognition and daily functioning. In the main stimulation approach, DBS leads implanted in the ventral anterior limb of the internal capsule and adjacent ventral striatum (VC/VS) are intended to modulate the same prefrontal cortex-basal ganglia-thalamic circuitry targeted by lesion procedures. Related approaches include targeting VS subterritories (nucleus accumbens or ventral caudate) using DBS.

Outcomes data are mainly open-label, though small studies have used sham-controlled DBS. In one case a sham gamma knife lesion was compared to actual ablations in the VC/VS, in the first controlled trial of a lesion procedure for any psychiatric illness. Across procedures, meaningful benefit appeared in 30-60% when assessed after varying delays. The longstanding clinical impression that exposure-based behavioral therapies (exposure and response prevention, or ERP) can become more effective after vs. before neurosurgery has continued. This has led to explicit incorporation of ERP early in the post-surgery course in at least one study.

Mean severity decreases from “very severe” to “moderate” illness were usually but not always plleled by functional gains. Given that the chronically severe symptoms in these patients are profoundly disruptive to the life course, capturing possible changes in functioning might require a year-long period of observation, even if symptom lessening appears earlier. The prognostic significance of differences in OCD clinical phenomenology to surgical outcomes is beginning to be addressed. Ego-dystonic OCD symptoms primarily based in harm avoidance might improve most, although symptoms of “incompleteness” (motivated by an intense desire to achieve a “just right” feeling) have lessened in some patients as well.

Translational research mapping ventral prefrontal-basal circuitry in primates has delineated potential pathways of DBS effect. Stimulation-related changes in behavioral phenotypes related to fear conditioning and extinction are the focus of ongoing collaborative work, in concert with our controlled multicenter trial of VC/VS DBS for intractable OCD. For OCD sufferers as a whole, the major promise of this line of research is that better delineation of the neurocircuit and behavioral mechanisms necessary to therapeutic benefit will have application using less invasive treatment strategies.

Dr. Anthony A. Grace from the Departments of Neuroscience, Psychiatry and Psychology at the University of Pittsburgh presented a talk entitled “Animal Models of OCD: Frontal Cortical Disruption and Modulation by DBS.” In this talk Dr. Grace covered what we have found about the mechanisms of deep brain stimulation (DBS) action and how it can relate to therapy in humans. An issue in understanding DBS is a better understanding of its mechanism of action. DBS has been carried out in the treatment of neurological disorders by stimulating regions that previous studies have targeted for lesions to achieve a therapeutic action. This gave rise to the “functional lesion” hypothesis of DBS action. However, the stimulation frequency typically used for DBs is 130 Hz, which for neurophysiologists typically is associated with long-term potentiation rather than inhibition. Therefore, we examined the effects of DBS using the ventral striatal circuit that is typically used for the treatment of Obsessive Compulsive Disorder (OCD) in humans.

OCD is typically associated with hyperactivity in frontal cortical structures, notably the orbitofrontal cortex. We found that DBS of the ventral striatum for 90 minutes caused a decrease in the firing rate of orbitofrontal neurons, along with firing through antidromic activation of a small percentage of the pyramidal output neurons in this area. In contrast, when we presented single stimuli, we found that there was a classical NMDA-dependent long-term potentiation of the evoked field potential, which is a recording of the total electrical signal originating in the orbitofrontal cortex caused by the stimulation. Moreover, interneurons in the cortex were found to be more responsive to stimulation. This led to the conclusion that DBS caused antidromic activation of a small number of pyramidal neurons and, via local axon collaterals, long-term potentiation of the excitation of interneurons. Therefore, the DBS was not causing a “functional lesion,” but instead caused activation of interneurons to increase inhibition within the orbitofrontal cortex. This is consistent with the concept that the overactivity in the orbitofrontal cortex may have been due to deficits in interneuron inhibition.

Besides inhibiting pyramidal neurons, interneurons have another important function within the cortex and other structures - they control the generation of rhythmic activity within these structures. Rhythmic activity is believed to represent the functional state of a region, with beta and gamma oscillations representing neuronal activation that correlates with increases in metabolism seen with functional imaging. In awake animals, the effects of DBS are transient in nature, wearing off minutes after turning off the stimulation; this suggests that the brain is organized in a manner to “push back” from the effects of DBS. However, when the stimulation is done for at least 5 days, there are significant changes in these frequencies. First, the short-term increases in power seem to give way to changes in coherence, or the organization of activity between regions. In particular, the orbitofrontal cortex showed increased coherence between itself and other regions, suggesting that the orbitofrontal cortex was now more responsive to inputs. This is consistent with the functional pathology of OCD - i.e., the orbitofrontal cortex is responsible for devaluing a stimulus; something that OCD patients fail to do. Therefore, when an OCD patient finds that a door is locked, instead of devaluing its significance in the face of data, their orbitofrontal cortex keeps “reminding” them that the door may indeed be unlocked. By restoring the ability of the orbitofrontal cortex to respond to this information, DBS may enable the patient to “let go” of inappropriate stimuli in the face of evidence to the contrary.

### 2C. Trauma and fear

Dr. Roger Pitman a Professor of Psychiatry at Massachusetts General Hospital and Harvard Medical School, Boston, MA, USA delivered a lecture entitled, “Stress, Anxiety, and Trauma.” Because of the breadth of this topic, Dr. Pitman focused on his group’s research into biological aspects of posttraumatic stress disorder (PTSD). He noted that although PTSD is still largely regarded as a psychological phenomenon, ultimately the impact of an environmental event, such as a psychological trauma, must be understood at organic, cellular, and molecular levels. Dr. Pitman reviewed research in the psychophysiology laboratory beginning in the 1980’s indicating that trauma victims diagnosed with PTSD showed larger heart rate, skin conductance, and facial electromyogram responses during personal, script-driven imagery of their traumatic events. When the script-driven imagery method was taken into the positron emission tomography (PET) scanner, results indicated that PTSD subjects also showed greater concurrent activation of amygdalocentric anterior plimbic brain areas thought to underlie emotional experience. However, one brain region, the ventromedial prefrontal cortex (vmPFC), showed pdoxically reduced activation in PTSD subjects during traumatic imagery. Further work with functional magnetic resonance imaging (fMRI) revealed an inverse association between vmPFC and amygdala activation, suggesting that vmPFC activation acts to inhibit amygdala and thereby confers resilience.

Pitman also presented results of a 15-year ongoing study of identical twins discordant for combat exposure in Vietnam, which has addressed the origin of biological abnormalities in PTSD. If his combat-unexposed co-twin shares an abnormality in a PTSD veteran, it is unlikely to have been acquired as a result of combat trauma but rather likely represents a familial vulnerability factor. If it is not shared, it is likely to have been acquired. This research has suggested that greater heart rate response to loud (startling) tones, lower electroencephalographic P300 event-related potential (ERP) response, increased P2 intensity dependence, lower vmPFC volume according to voxel-based morphometry, and impaired retention of extinction of a de novo conditioned fear response are acquired PTSD biomarkers, whereas more neurological soft signs (suggestive of subtle central nervous system impairment), diminished volume of the hippocampus, and both hypermetabolism and hyperreactivity of dorsal anterior cingulate cortex (a brain region found to promote the fear response) are familial vulnerability factors for PTSD.

Dr. Mohammed R Milad from the Department of Psychiatry at the Department of Psychiatry and Massachusetts General Hospital presented a talk entitled “Neurobiology of learning not to fear: implications to PTSD and beyond”. In his presentation, Dr. Milad presented data on the neural correlates of fear extinction using a translational and multi-modal approach. After a brief definition and description of the classical fear conditioning pdigms used to study fear extinction, Dr. Milad presented data describing the basic mechanisms of how fear extinction learning is formed and stored with a network of brain regions including the ventromedial prefrontal cortex (vmPFC), the amygdala and the hippocampus. Dr. Milad presented data regarding the apparently apposing effects of two prefrontal regions on fear expression: the activation of the dorsal anterior cingulate cortex (dACC) seems to enhance the expression of conditioned fear, and the vmPFC seems to quell the expression of fear. The function of these brain regions within the context of fear learning and extinction seems to be impaired in patients diagnosed with PTSD, and that such functional impairment seem to be associated with exaggerated fear expression in PTSD patients.

In the second part of his talk, Dr. Milad described the potential role of gonadal hormones, such as estrogen, on fear extinction and how these hormones may contribute to sex differences in fear learning and extinction and may also contribute to the differences in the epidemiology of PTSD between men and women. In brief, Dr. Milad presented data showing that an increased estrogen level in both female rats and in women is associated with enhanced consolidation of extinction memory. The elevated levels of estrogen seem to interact with the functional activation of the fear extinction network, specifically, the higher the estrogen levels, the more activation the vmPFC exhibits during extinction recall in women. The importance of assessing hormonal status in women during treatment for PTSD and other anxiety disorders was highlighted in the presentation.

Dr. Gregory Quirk from the University of Puerto Rico presented a talk entitled “Neurobiological Basis of Conditioned Fear and Extinction” in which he talked about the neural mechanism of fear conditioning, using rats as a model organism. Fear conditioning has become a popular model of emotional learning, in which neurons in the amygdala, hippocampus, and prefrontal cortex acquire information about stimuli (such as tones) that predict danger (such as shocks). Disruption of such circuits leads to excessive fear seen in anxiety disorders such as PTSD and OCD. Quirk showed that expression of fear in the amygdala is under bidirectional control by the prelimbic and infralimbic subdivisions of prefrontal cortex. Modulation of activity in these circuits can increase fear either by facilitating expression or by impairing extinction of fear. In addition to modulating fear (such as freezing), these areas also modulate avoidance behaviors, in which rats escape a shock by moving to a safe place. Deep brain stimulation (DBS) of the ventral striatum has been shown to help some patients suffering from OCD, and Quirk showed rodent data suggesting that DSB may act by augmenting plasticity in extinction circuits located in amygdala and prefrontal areas. In collaboration with Dr. Haber, Quirk is characterizing the striatal-cortical circuits by which DBS enhances extinction of fear.

Topic 3: Anxiety and depression: perspective from the region.

Dr. Abdul-Monaf Al-Jadiry, Professor and chair of Psychiatry, Department of Medicine, Medical Faculty, The University of Jordan, Amman, Jordan gave a presentation entitled, "Prevalence of Anxiety and Mood Disorders and the link to Trauma: An Iraqi Perspective". The presentation initiated with a general conclusion, based on literature review, about the impact of trauma on mental health in general, and the prevalence of anxiety and mood disorders in particular. Dr Al-Jadiry presented data from Iraqi Mental Health Survey (IMHS) to demonstrate the link between the two. IMHS is the first nationwide mental health epidemiological survey in Iraq and the second in the Middle East after Lebanon. The IMHS was undertaken in the years 2006-207, amidst circumstances of extreme instability and minimal security following the second gulf war. IMHS used World Mental Health Survey (WMHS) methodology and diagnostic instrument to provide data on the prevalence of mental health disorders, their impact and treatment, and their relationship to violence in the population. Trained interviewers used the “Composite International Diagnostic Interview (CIDI), Version 3” as a diagnostic tool for the assessment of mental disorders. The IMHS for the first time provides evidence-based data about the prevalence of mental health problems and mental health of Iraqi people. The survey provides an initial indicator of lifetime prevalence, and 12 months and 30 days prevalence rates alongside the experience of trauma. The importance of the Iraqi example springs from the fact that the state of Iraqis has been unique, as they have suffered more than any other people from decades of war trauma, sanctions and violence, which are still going on.

The IMHS demonstrated that 11.58% of population suffered from any anxiety disorder, including stress disorder, lifetime, and 7.82% from any affective disorder lifetime, with a significant difference between women and men, age groups, and several other socio-demographic variables. Moreover, the overall lifetime exposure to traumatic events, according to the survey was 60.02%. In the lifetime prevalence rate of these disorders cases had significant higher exposure to trauma. The most striking finding of the survey is the very high exposure of the population to various types of trauma and the relative low prevalence rates of anxiety and mood disorders though in line with prevalence rates reported in Lebanon and Afghanistan. Dr. Al-Jadiry attributed this discrepancy to the biological nature of people vulnerability for anxiety and mood disorders in addition to inherent protective sociocultural characteristics of Iraqis; these include: development of resilience, high community support, rituals to canalize distress and bereavement in a socially acceptable manner, and viewing losses as divine states. Moreover, the effect of stigma of mental illness is to make some patients hide their symptoms. Dr Al–Jadiry concluded, despite massive exposure to traumatic events, the prevalence of anxiety and mood disorders amongst Iraqis is relatively lower than expected, though in accordance with the literature from post conflict zones.

Dr. Walid Sarhan a consultant Psychiatrist from Amman Jordan gave a talk entitled “Cognitive behavioral therapy of Islamic religious OCD”. The talk started by brief background of Islamic religion with emphases on how its comprehensive application to people’s daily lives and its rules to be followed in praying, washing, cleaning may relate to OCD symptoms expressed in some of the patients in the region. In managing those patients, Dr. Sarhan discussed how therapists may need to be aware of the compatibility of the cognitive behavioral therapy with the Islamic religion. The clinical experience of dealing with cleaning, abolition, praying, divorce and doubts in beliefs were discussed in light of the literature, while at the same time addressing the common clinical challenges presented by Arab Muslim patients suffering from OCD. Dr. Sarhan discussed how religious individuals with authority to declare legal rulings or decrees (Fatwa) on some aspects of the Islamic religion may also influence patient’s symptoms. The clinician’s knowledge of some of these rulings or “fatwas” may be necessary to understanding how some aspects of the OCD symptoms (especially those related to cleansing) came to exist.

In obsessions related to one’s faith, Dr. Sarhan suggested that clinician’s knowledge of the Islamic religion might be used as an additional tool to help aid the treatment; a clinician could make use of the teachings of the prophet to his followers as the key element in cognitive therapy.

Dr. Radwan A. BaniMustafa from the University of Jordan Medical School & Hospital gave a talk entitled “Depression in Primary patients with chronic medical diseases” he described a study which included a group of 407 primary care patients with chronic medical diseases. These patients were seen in the primary care clinics in Jordan University hospital in 2011. Prevalence of depression in this sample was 45.5%. Depression was significantly associated with young age, low level of education, chronic headache and chronic musculoskeletal diseases. In addition, it was associated but not significantly so with having more than three medical problems, chronic cardiopulmonary disorders, chronic Skin and thyroid disorders, female sex and single status.
